# Comparative evaluation of weight-bearing cone beam CT arthrography and supine 3T MRI in knee osteoarthritis

**DOI:** 10.1016/j.ostima.2025.100382

**Published:** 2025-11-12

**Authors:** Antti Kemppainen, Vilja Kotkaranta, Olli Nykänen, Mika T. Nevalainen

**Affiliations:** a Research Unit of Health Sciences and Technology, Faculty of Medicine, University of Oulu, P.O. Box 5000, FI-90014, Oulu, Finland; b Department of Diagnostic Radiology, Oulu University Hospital, P.O. Box 50, FI-90029, Oulu, Finland; c Department of Applied Physics, Faculty of Science and Forestry, University of Eastern Finland, Kuopio, Finland; d Medical Research Center Oulu, University of Oulu and Oulu University Hospital, Oulu, Finland

**Keywords:** Arthrography, Cone-beam computed tomography, Knee joint /diagnostic imaging, Magnetic resonance imaging, MOAKS, Osteoarthritis /diagnostic imaging

## Abstract

**Objective:**

To evaluate the agreement of cone-beam computed tomography arthrography (CBCTA) and magnetic resonance imaging (MRI) in detecting osteoarthritic changes of the knee.

**Design:**

This comparative study included 58 knee joints in 54 symptomatic subjects with suspicion of knee osteoarthritis (OA). The symptomatic joints were imaged using CBCTA and 3T MRI and graded using the MRI Osteoarthritis Knee Score (MOAKS). Agreement between modalities was assessed using prevalence and bias adjusted kappa (PABAK), percentages of exact (PEA) and close agreement (PCA) of ±1 in MOAKS grades and participant-specific comparisons.

**Results:**

CBCTA was performed with acceptable intra-articular concentration in 86.2 % (*n* = 50) knees in 48 subjects (68.6 % women, mean age 58.7 years). Definite tibiofemoral and patellofemoral OA was identified on MRI in 76 % (*n* = 38). For all cartilage lesions, PABAKs ranged between 0.80 and 0.96 (mean 0.90), with mean PEAs of 68.4 % and mean PCAs of 90.2 %. Full-thickness cartilage lesions demonstrated particularly strong agreement. Osteophyte detection yielded PABAKs between 0.92 and 0.98 (mean 0.95), mean PEA of 65.8 % and mean PCA of 99 %. For meniscal pathology, PABAKs ranged from 0.84 to 0.98 (mean 0.90), with mean PCA of 74.7 % and mean PEA of 81.7 %. For the anterior cruciate ligament, Baker cyst, and synovial hypertrophy, PABAKs were 0.97, 0.63, and 0.65, with high PEAs.

**Conclusions:**

CBCTA demonstrates moderate to almost perfect agreement with 3T MRI for knee OA findings. Although 13.8 % of the arthrographies had failed in our study, CBCTA offers a practical alternative when MRI is contraindicated or unavailable.

## Introduction

Knee osteoarthritis (OA) is a common degenerative disease with increasing prevalence worldwide [1]. OA is characterized by cartilage damage, synovial inflammation, osteophyte formation, remodeling of the subchondral bone and, ultimately, destruction of the joint. The radiological diagnosis of knee OA is usually acquired by conventional radiographs, where either clear radiographic OA (osteophyte formation, joint space narrowing and subchondral bone changes) or lack of alternative diagnoses is usually expected. Other imaging modalities, mainly magnetic resonance imaging (MRI) and computed tomography (CT), are occasionally requested for a more detailed evaluation [2,3]. Due to its high sensitivity and excellent soft tissue contrast, MRI is useful in detecting early signs of OA such as cartilage damage, associated bone marrow lesions, osteophyte formation, meniscal degeneration, effusion and synovitis, and is the most common imaging modality in OA research [4]. CT is excellent at assessing osseous structures, subchondral bone changes [5] and tissue mineralization [6] with much cheaper and faster imaging than MRI. CT, however, provides inferior soft tissue contrast and involves exposure to ionizing radiation [3].

Cone-beam CT (CBCT) scanners have become frequent in orthopedic clinics during the 21st century [7], offering high resolution extremity CT data with substantially less radiation, requires less space and less initial investment as well as lower maintenance costs compared to multi-row detector CT [[7], [8], [9]]. Multiple CBCT systems are able to image joint structures in weight-bearing, allowing, for example, quantitative evaluation of joint space width in axial loading in the knee joint [10]. Accordingly, clinical CBCT use has been well established for the foot, ankle, knee and hip joints, as well as the hand and elbow joints [7,9,11]. Where applicable, CBCT of the knee joint is imaged in standing position with the knee in a fixed flexion position and standardized positioning [11]. Knee flexion angle and quadriceps muscle activity are significant factors in patellar tracking. The typical radiologic measurements of patellofemoral maltracking are significantly changed in the standing position in CBCT as well as in MRI [[11], [12], [13], [14]], but the standing position is not possible in common MRI systems. CBCT has also been found to be reliable and reproducible for evaluating implant rotation and loosening in total knee arthroplasty patients [[15], [16], [17], [18]].

CT arthrogram of the knee requires an iodinated contrast agent injected into the knee joint, followed by a CT scan. Although the contrast resolution of soft tissue structures in CT in general is poor, the intra-articular contrast agent envelops most of the relevant soft tissue structures. Joint pathology is then diagnosed by identifying anatomic discrepancies and discontinuities in the intra-articular filling of the contrast agent. Although use of CT arthrography is low compared to MRI, CT arthrography offers decent tissue contrast and has been found to be useful in detecting cartilage defects [19], meniscal tears and ACL injuries [20]. It also has potential as a diagnostic modality for open knee injuries [21]. In fact, CT arthrography is considered the reference imaging modality for cartilage lesions [3] compared to 1.5T MRI [19,22]. Compared to CT arthrography, CBCT arthrography (CBCTA) has been investigated substantially less [3,23]. The relative attenuation of structurally normal and damaged articular cartilage has been evaluated previously in CBCTA [24,25] and a recent study reported overall lower performance for 1.5T MRI in detecting cartilage lesions compared with CBCTA as the reference standard [23].

The diagnostic capability of CT arthrography of the knee in assessing OA-associated findings has been previously demonstrated. CBCT offers diagnostic, radioprotective and financial advantages over conventional CT equipment. CBCTA also appears to outperform supine 1.5T MRI in detecting cartilage lesions. However, 3T MRI offers higher image resolution and superior diagnostic performance compared to knee arthroscopy [26]. Thus, the purpose of this study was to evaluate the agreement of weight-bearing CBCTA of the knee joint in detecting OA-associated findings evaluable in MRI, using the MRI Osteoarthritis Knee Score (MOAKS) [[27], [28], [29]] system for comparison.

## Method

The study was conducted with approval of the local institutional review board in accordance with an approved ethical statement. All participants provided informed consent (Northern Ostrobothnia Hospital District Ethical Committee, permission numbers 88/2019 and 144/2019).

### Study population

This was single-center comparative study, in which 58 knee joints in 54 volunteer subjects with knee symptoms were imaged using supine MRI and weight-bearing CBCTA between March 2022 and August 2022. Exclusion criteria were suspicion of acute infection, fractures or tumors in the vicinity of the knee joint, rheumatic disease, contraindications to MRI and allergies to iodine contrast media. In case of bilateral symptoms (7.4 %, *n* = 4), both knees were imaged. After imaging, CBCTAs were preliminarily evaluated by the reader (MTN); knee joints with low intra-articular contrast agent concentration or significant contrast material extravasation were excluded (13.8 %, *n* = 8). In total, 50 knee joints with complete and technically well performed CBCTAs (and MRIs) were analyzed.

### MRI and CBCTA imaging

For MRI, Siemens Vida 3T scanner (Siemens Healthineers, Erlangen, Germany) was used with a standard knee coil and identical MRI protocol for all knee joints. The protocol included two isotropic SPACE sequences (Sampling perfection with application optimized contrast using different flip angle evolution): a T2-weighted fat-saturated sequence (repetition time, 1000 ms; echo time, 125 ms) and a PD-weighted sequence (repetition time, 900 ms; echo time, 76 ms). Both sequences used a 448 × 448 matrix, 0.40 × 0.40 mm in-plane resolution, 140 × 180 mm² field of view, and 0.80 mm slice thickness and spacing.

CBCTA was done the day after knee MRI for all participants using a Planmed Verity extremity CBCT scanner (Planmed, Helsinki, Finland). For the preceding arthrography, knee joints were injected with 6 ml Omnipaque 300mg/ml + 14 ml NaCl using either a lateral suprapatellar or medial tibiofemoral approach with the knee joint in slight flexion [30]. All injections were administered with a 22-gauge needle and ultrasound guidance while using sterile technique. After the injection, participants performed several knee flexion-extensions while sitting. The subsequent weight-bearing CBCT was done in a single-leg stance to ensure full weight-bearing on the imaged knee joint. Routine settings were used: tube voltage 96 kV, tube current 8 mA, slice thickness and spacing 0.4 mm, field of view 160 × 160 mm^2^, pixel spacing 0.4 × 0.4 mm and a 401 × 401 matrix.

### Image analysis

The semi-quantitative MOAKS [[27], [28], [29]] classification system with identical grading schemes was used to grade both MRI and CBCTA images (blinded to modality) by a fellowship-trained musculoskeletal radiologist with 11 years of experience (MTN). Both modalities were assessed in random order with a two-week washout between the readings. Cartilage lesions, osteophytes, menisci, cruciate ligaments and the presence of Baker cyst and synovial hypertrophy were evaluated in this study. Cartilage lesions of all severities and full-thickness cartilage lesions were graded separately in 14 MOAKS-defined subregions as: 0 (none), 1 (<10 % of the subregional area affected), 2 (10–75 %), 3 (>75 %). Osteophytes were graded in 12 locations as: 0 (none), 1 (small), 2 (moderate), 3 (large). The medial and lateral meniscus was graded in thirds, and the anterior, central and posterior third was graded separately as: 0 (normal), 1 (intrameniscal signal), 2 (vertical tear), 3 (horizontal tear), 4 (complex tear), 5 (partial maceration), 6 (total maceration). The cruciate ligaments were graded as intact (0) or torn (1). Baker cyst and synovial hypertrophy were graded as absent (0) or present (1). Synovial hypertrophy was evaluated regardless of joint effusion, although both are frequently present in OA [28]. On MRI, synovial hypertrophy was regarded as definite thickened synovial tissue [28] and on CBCTA as definite amorphous lower Hounsfield unit (HU) material compatible with synovial thickening. Bone marrow lesions, meniscal extrusion, meniscal hypertrophy as well as OA-associated features not included in the MOAKS system such as joint space narrowing and subchondral sclerosis were not assessed.

### Statistical methods

Count data are presented as number of observations (n) and as a percentage of the total knee joints analyzed (*n* = 50). The number of excluded CBCTAs is presented as count (n) and as a percentage of imaged knee joints before exclusion (*n* = 58). Participant age is reported as mean and standard deviation. In describing imaging findings, medial and lateral patellar and anterior femoral MOAKS-defined cartilage subregions, as well as medial and lateral patellar and trochlear (anterior femoral) osteophytes, were classified as part of the patellofemoral joint. Medial and lateral central and posterior femoral cartilage subregions and osteophytes as well as all tibial cartilage subregions and osteophytes were classified as part of the tibiofemoral joint.

To describe the overall imaging findings in the study population, the prevalence of definite tibiofemoral and patellofemoral OA in the study population were approximated using a proposed consensus MRI definition by Hunter et al. [31]. Tibiofemoral OA was defined as either 1) any tibiofemoral osteophyte MOAKS ≥ 2 plus any tibiofemoral full-thickness cartilage loss, or 2) either one of the previous findings and two of the following: horizontal meniscal tear, meniscal maceration, any tibiofemoral cartilage loss MOAKS ≥ 2. Patellofemoral OA was defined as any patellofemoral osteophyte MOAKS ≥ 2 plus either any patellofemoral cartilage loss MOAKS ≥ 2 or any patellofemoral full-thickness cartilage loss.

To quantify the inter-method agreement, MOAKS grades on MRI and CBCTA were compared using prevalence and bias adjusted kappa (PABAK), percentages of exact and close agreement and participant-specific comparisons. PABAKs were interpreted as in [32]: <0.2 was regarded as poor agreement between imaging modalities; 0.21–0.39 as minimal; 0.40–0.59 as weak; 0.60–0.79 as moderate; 0.80–0.90 as strong; and higher than 0.90 as almost perfect agreement. The percentage of exact agreement (PEA) was defined as the percentage of identical CBCTA and MRI MOAKS grades for each evaluated structure, and the percentage of close agreement (PCA) was defined as the percentage of identical or ±1 difference in CBCTA and MRI MOAKS grades. For participant-specific comparisons, we reported the number of MOAKS-defined subregions with higher or lower grade on MRI compared to CBCTA (MRI > CBCTA and MRI < CBCTA, respectively), grade 0 in MRI and grade other than 0 in CBCTA (MRI-, CBCTA+), the reverse (MRI+, CBCTA–) and the number of participants with either grade 0–1 or 1–2 on both examinations were reported for cartilage lesions, full-thickness cartilage lesions and osteophytes. The same comparisons were made for other evaluated structures, except for the last comparison.

## Results

**Fig. 1:**
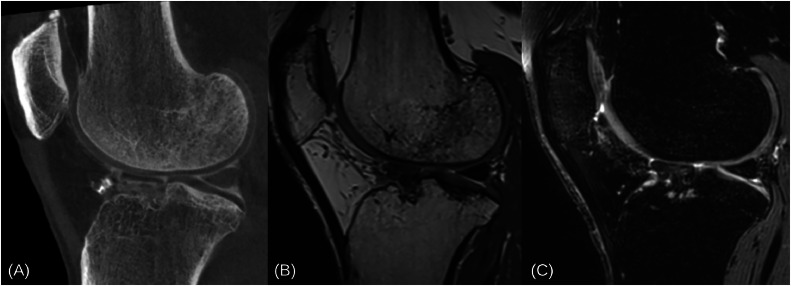
A technically successful CBCT arthrography (A) of the knee joint in a 46-year-old man in sagittal orientation. Intra-articular contrast agent delineates patellofemoral and tibiofemoral cartilage surfaces and reveals the menisci and other intra-articular soft tissue structures (A) similarly to PD-weighted (B) and T2-weighted fat-saturated (C) MRI sequences.

CBCTA achieved acceptable intra-articular concentration in 86.2 % (*n* = 50) knee joints in 48 participants (68.6 % women, mean age 58.7 ± 7.6 years) (Fig. 1). A total of 13.8 % (*n* = 8) knees were excluded due to either low concentration of intra-articular contrast or significant contrast extravasation on CBCT. The total radiation dose was similar in all CBCTA examinations with total volume CT dose index of 4.89 mGy, total dose-length product of 63.42 mGy*cm and total dose area product of 1343.87 mGy*cm^2^. All MRIs were performed completely and with the expected image quality (data not shown).

### Osteoarthritic imaging findings were common in the study population and found in similar proportions on CBCTA and MRI

**Fig. 2:**
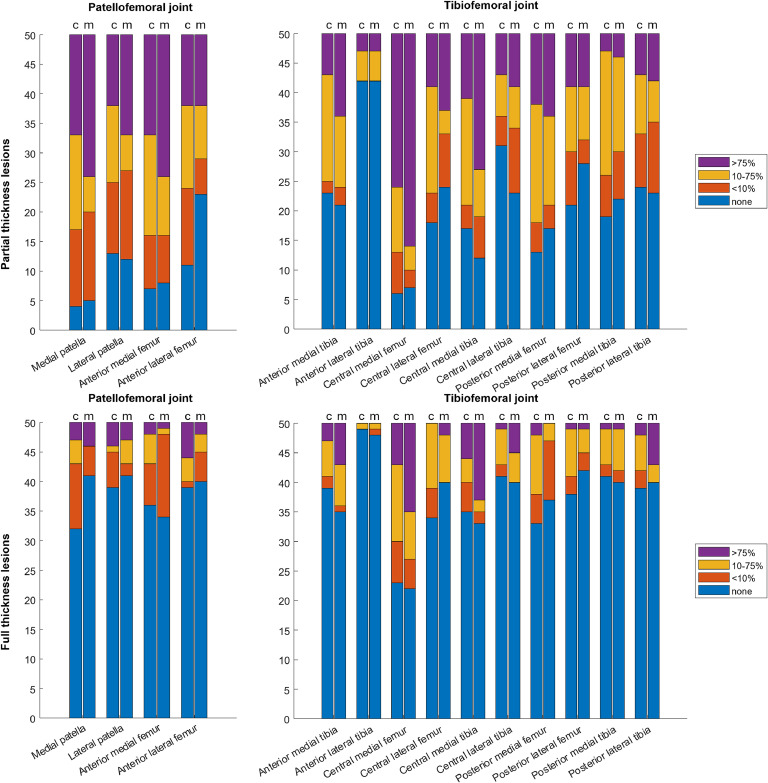
Counts of the CBCTA- (left bars, c) and MRI-detected (right bars, m) patellofemoral and tibiofemoral cartilage lesions and full-thickness cartilage lesions of different sizes in MOAKS-defined subregions.

**Fig. 3:**
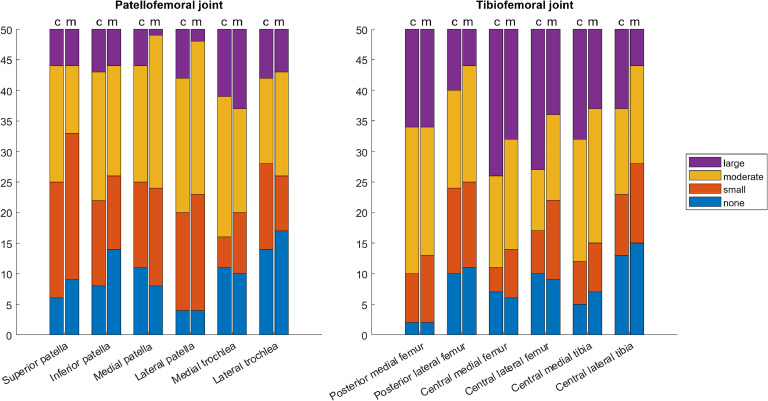
Counts of the CBCTA- (left bars, c) and MRI-detected (right bars, m) osteophytes of different sizes in MOAKS-defined subregions.

**Fig. 4:**
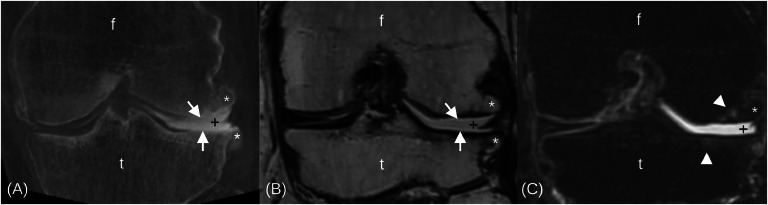
Lateral tibiofemoral osteoarthritis in a 60-year-old man in coronal orientation. CBCTA (A) depicts the full-thickness cartilage lesions of posterolateral femoral and tibial cartilage surfaces (arrows) with large tibiofemoral osteophytes (asterisks) as well as the absent, macerated, posterolateral meniscus (plus signs). PD- (B) and T2-weighted fat-saturated (C) MRI sequences confirm the CBCTA findings and show small cartilage lesion-associated subchondral bone marrow lesions (arrowheads).

Descriptive statistics for cartilage lesions, osteophytes and other evaluated structures in CBCTA and MRI are shown in Fig. 2, Fig. 3 and in Supplementary Tables S1-S3. Overall, cartilage lesions were frequent: MRI-normal cartilage subregions ranged from 10–46 % in the patellofemoral and 14–84 % in the tibiofemoral joint, with similar normal proportions on CBCTA (Fig. 2, Table S1). Full-thickness cartilage lesions were found less often but in similar proportions in both modalities. Severe grade 3 patellofemoral cartilage lesions were most often found in the medial patellar and the anterior medial femoral subregions with identical proportions for both modalities: 48 % of MRIs and 34 % of CBCTAs. In the tibiofemoral joint, the central medial femoral and tibial cartilage subregions were most often affected: the femoral subregion had a grade 3 lesion on 72 % of MRIs and 52 % of CBCTAs, and the tibial subregion had a grade 3 lesion in 46 % of MRIs and 22 % of CBCTAs. (Fig. 2, Fig. 4, Table S1)

Osteophytes were also frequent in the study population, shown in Fig. 3 and Table S2. Large osteophytes were most often found in the medial trochlear position (26 % of MRIs and 22 % of CBCTAs) and central medial and lateral femoral position (36 % and 28 % of MRIs, and 48 % and 46 % of CBCTAs. (Table S2)

Evaluations of meniscal morphology, cruciate ligaments and the presence of Baker cyst and synovial hypertrophy are shown in Table S3. Most of the medial and lateral meniscal thirds were normal on both CBCTA and MRI. Most pathologic findings were found in the central and posterior third of the medial meniscus, with complex tears and partial macerations the most frequent abnormal findings in both modalities. ACLs were graded as torn in two MRIs and three CBCTAs. PCL tears were absent. Baker cysts were identified in similar proportions between imaging modalities. Synovial hypertrophy was identified in 14 % of CBCTAs and 32 % of MRIs. (Fig. S1, Table S3)

The incidence of definite OA as defined by Hunter et al[31]. was high in the study population. Seven (14 %) did not have definite OA, 8 % (*n* = 4) had isolated tibiofemoral OA, one had isolated patellofemoral OA and 76 % (*n* = 38) had both tibiofemoral and patellofemoral OA based on MRI.

### Inter-modality comparison shows strong to almost perfect agreement between CBCTA and 3T MRI

The agreement between CBCTA and MRI is shown in Tables 1–4 and illustrated in Fig. 2, Fig. 3. In evaluating cartilage lesions, the diagnostic performance of CBCTA ranged from strong to almost perfect agreement, with weighted PABAKs varying between 0.80 - 0.95 (mean 0.87), PEAs between 46 and 90 % (mean 58.3 %) and PCAs between 80 and 98 % (mean 90.2 %) (Table 1). For full-thickness cartilage lesions only, PABAKs ranged between 0.88–0.96 (mean 0.92), PEAs between 66 and 94 % (mean 78.4 %) and PCAs between 84 and 98 % (mean 92 %) (Table 2). In all cartilage subregions, PEAs were higher for full-thickness cartilage lesions than for cartilage lesions in general, with an increase of 4–34 percentage points (mean 18.8 points). (Tables 1–2).

**Table 1:** Prevalence- and bias-adjusted kappas with 95 % Confidence Intervals, percentages of exact and close agreement and participant-specific inter-modality comparisons between knee CBCTA and MRI in evaluating articular cartilage lesions. PABAK; Prevalence and bias adjusted kappa, PCA; Percentage of close agreement, PEA; Percentage of exact agreement.

	PABAK	PEA	PCA	MRI > CBCTA	MRI < CBCTA	MRI-, CBCTA+	MRI+, CBCTA-	Both 0–1 or 2–3
	Cartilage lesions							
**Patellofemoral joint**								
Medial patella	0.88 (0.78 – 0.99)	50 %	94 %	13/50	12/50	4/50	3/50	39/50
Lateral patella	0.80 (0.67 – 0.93)	46 %	86 %	15/50	12/50	6/50	7/50	36/50
Anterior medial femur	0.87 (0.76 – 0.98)	52 %	90 %	16/50	8/50	4/50	3/50	40/50
Anterior lateral femur	0.91 (0.81 – 1)	62 %	92 %	3/50	16/50	12/50	0/50	43/50
**Tibiofemoral joint**								
Anterior medial tibia	0.82 (0.69 – 0.94)	56 %	80 %	15/50	7/50	5/50	7/50	39/50
Anterior lateral tibia	0.95 (0.87 – 1)	90 %	96 %	2/50	3/50	1/50	1/50	48/50
Central medial femur	0.90 (0.80 – 1)	60 %	94 %	14/50	6/50	3/50	2/50	45/50
Central lateral femur	0.82 (0.70 – 0.95)	54 %	82 %	10/50	13/50	10/50	4/50	38/50
Central medial tibia	0.81 (0.68 – 0.94)	48 %	84 %	19/50	7/50	4/50	9/50	38/50
Central lateral tibia	0.91 (0.81 – 1)	64 %	94 %	13/50	5/50	2/50	10/50	44/50
Posterior medial femur	0.86 (0.75 – 0.97)	58 %	86 %	8/50	13/50	6/50	2/50	39/50
Posterior lateral femur	0.87 (0.76 – 0.98)	62 %	86 %	5/50	14/50	11/50	4/50	42/50
Posterior medial tibia	0.83 (0.70 – 0.95)	54 %	80 %	9/50	14/50	8/50	5/50	36/50
Posterior lateral tibia	0.90 (0.80 – 1)	60 %	94 %	11/50	9/50	5/50	6/50	42/50

**Table 2:** Prevalence- and bias-adjusted kappas with 95 % Confidence Intervals, percentages of exact and close agreement and participant-specific inter-modality comparisons between knee CBCTA and MRI in evaluating full-thickness articular cartilage lesions. FT; Full-thickness, PABAK; Prevalence and bias adjusted kappa, PCA; Percentage of close agreement, PEA; Percentage of exact agreement.

	PABAK	PEA	PCA	MRI > CBCTA	MRI < CBCTA	MRI-, CBCTA+	MRI+, CBCTA-	Both 0–1 or 2–3
	FT Cartilage lesions							
**Patellofemoral joint**								
Medial patella	0.93 (0.84 – 1)	68 %	94 %	4/50	12/50	12/50	3/50	47/50
Lateral patella	0.96 (0.90 – 1)	80 %	98 %	4/50	6/50	5/50	3/50	48/50
Anterior medial femur	0.92 (0.83 – 1)	72 %	94 %	7/50	7/50	5/50	7/50	45/50
Anterior lateral femur	0.94 (0.86 – 1)	84 %	92 %	1/50	7/50	2/50	1/50	45/50
**Tibiofemoral joint**								
Anterior medial tibia	0.90 (0.80 – 1)	76 %	88 %	10/50	2/50	2/50	6/50	43/50
Anterior lateral tibia	0.94 (0.86 – 1)	94 %	96 %	2/50	1/50	1/50	2/50	48/50
Central medial femur	0.89 (0.78 – 0.99)	64 %	90 %	13/50	5/50	4/50	5/50	43/50
Central lateral femur	0.96 (0.89 – 1)	82 %	96 %	3/50	6/50	6/50	0/50	47/50
Central medial tibia	0.87 (0.76 – 1)	84 %	86 %	7/50	1/50	1/50	3/50	43/50
Central lateral tibia	0.91 (0.82 – 1)	84 %	92 %	6/50	2/50	2/50	3/50	45/50
Posterior medial femur	0.88 (0.77 – 0.98)	66 %	84 %	5/50	12/50	9/50	5/50	41/50
Posterior lateral femur	0.93 (0.85 – 1)	80 %	94 %	3/50	7/50	5/50	1/50	44/50
Posterior medial tibia	0.91 (0.82 – 1)	82 %	86 %	5/50	4/50	3/50	4/50	43/50
Posterior lateral tibia	0.96 (0.90 – 1)	82 %	98 %	7/50	2/50	2/50	1/50	48/50

For discrepant MOAKS cartilage grades between imaging modalities, the proportions of higher grades between modalities were similar in medial and lateral patellar cartilage regions. MRI tended to yield higher grades in the anterior medial femoral region and CBCTA in the anterior lateral femoral region. For the tibiofemoral cartilage regions, a trend of higher MRI grades was seen in the anterior and central medial tibial as well as central medial femoral and central lateral tibial subregions. A trend of higher CBCTA grades was seen in the posterior medial and lateral femoral and posterior medial tibial subregions. For all cartilage subregions, both imaging modalities produced false negative findings when compared to the other modality as the reference standard. The number of MRI-, CBCTA+ findings was noticeably higher compared to MRI+ CBCTA- findings in the anterior lateral femoral, central lateral femoral and posterior lateral femoral regions. The number of MRI+, CBCTA- findings was noticeably higher compared to MRI-, CBCTA+ findings in the central medial and lateral tibial subregions. The majority of MOAKS grades were either 0–1 or 2–3 for both imaging modalities in all subregions. (Table 1)

For full-thickness cartilage lesions, higher MRI grades compared to higher CBCTA grades were more frequent in the anterior medial tibial and central medial femoral and tibial subregions. Conversely the number of higher CBCTA grades compared to higher MRI grades was noticeably higher in medial patellar and posterior medial femoral subregions. MRI-, CBCTA+ findings were more frequent in the medial patellar and central lateral femoral subregions. Almost all gradings were either 0–1 or 2–3 for both imaging modalities. (Table 2)

For all osteophytes, the inter-modality agreement was almost perfect with PABAKs ranging between 0.92 - 0.98 (mean 0.95), PEAs between 52–84 % (mean 65.8 %) and PCAs between 98–100 % (mean 99 %) (Table 3). Of discordant MOAKS-grades, CBCTA had a general tendency to grade osteophytes higher than MRI. Few negative findings relative to the other modality were observed across all evaluated osteophyte regions for both MRI and CBCTA (Table 3).

**Table 3:** Prevalence- and bias-adjusted kappas with 95 % Confidence Intervals, percentages of exact and close agreement and participant-specific inter-modality comparisons between knee CBCTA and MRI in evaluating osteophytes. PABAK; Prevalence and bias adjusted kappa, PCA; Percentage of close agreement, PEA; Percentage of exact agreement.

	PABAK	PEA	PCA	MRI > CBCTA	MRI < CBCTA	MRI-, CBCTA+	MRI+, CBCTA-	Both 0–1 or 2–3
	Osteophytes							
**Patellofemoral joint**								
Superior patella	0.92 (0.83 – 1)	52 %	98 %	5/50	18/50	6/50	2/50	39/50
Inferior patella	0.94 (0.86 – 1)	58 %	100 %	5/50	16/50	7/50	1/50	46/50
Medial patella	0.93 (0.84 – 1)	56 %	98 %	10/50	12/50	4/50	7/50	43/50
Lateral patella	0.93 (0.85 – 1)	60 %	98 %	5/50	15/50	2/50	2/50	39/50
Medial trochlear	0.95 (0.88 – 1)	66 %	100 %	8/50	9/50	3/50	3/50	19/50
Lateral trochlear	0.98 (0.93 – 1)	84 %	100 %	3/50	5/50	3/50	0/50	46/50
**Tibiofemoral joint**								
Central medial femur	0.97 (0.92 – 1)	80 %	100 %	5/50	8/50	0/50	0/50	43/50
Central lateral femur	0.94 (0.87 – 1)	62 %	100 %	7/50	12/50	5/50	4/50	47/50
Central medial tibia	0.95 (0.87 – 1)	70 %	98 %	1/50	9/50	0/50	1/50	47/50
Central lateral tibia	0.94 (0.86 – 1)	66 %	98 %	3/50	16/50	2/50	3/50	45/50
Posterior medial femur	0.96 (0.90 – 1)	74 %	100 %	2/50	13/50	3/50	1/50	45/50
Posterior lateral femur	0.93 (0.86 – 1)	62 %	98 %	2/50	15/50	3/50	1/50	43/50

For the menisci, PABAKs ranged between 0.84 - 0.98 (mean 0.90), PEAs between 52–88 % (mean 74.7 %) and PCAs between 68–100 % (mean 81.7 %). For the ACL, Baker cyst and synovial hypertrophy, the PABAK values were 0.97, 0.63, and 0.65, the PEAs were 98 %, 72 %, 74 % and PCAs were 100 % as expected. PCL tears were absent (Table 4).

**Table 4:** Prevalence- and bias-adjusted kappas with 95 % Confidence Intervals, percentages of exact and close agreement and participant-specific inter-modality comparisons between knee CBCTA and MRI in evaluating meniscal morphology, cruciate ligaments, Baker cyst and synovial hypertrophy. ACL; Anterior cruciate ligament, PABAK; Prevalence and bias adjusted kappa, PCA; Percentage of close agreement, PCL; Posterior cruciate ligament, PEA; Percentage of exact agreement.

	PABAK	PEA	PCA	MRI > CBCTA	MRI < CBCTA	MRI-, CBCTA+	MRI+, CBCTA-
Anterior medial meniscus	0.84 (0.73 – 0.96)	84 %	84 %	1/50	7/50	7/50	1/50
Medial meniscus body	0.85 (0.74 – 0.96)	56 %	68 %	16/50	6/50	4/50	11/50
Posterior medial meniscus	0.86 (0.75 – 0.96)	52 %	68 %	19/50	5/50	2/50	11/50
Anterior lateral meniscus	0.96 (0.89 – 1)	88 %	90 %	4/50	2/50	2/50	3/50
Lateral meniscus body	0.93 (0.85 – 1)	80 %	86 %	9/50	1/50	1/50	6/50
Posterior lateral meniscus	0.98 (0.93 – 1)	88 %	94 %	3/50	3/50	3/50	0/50
ACL tear	0.97 (0.92 – 1)	98 %	100 %	0/50	1/50	1/50	0/50
PCL tear	N/A						
Baker cyst	0.63 (0.46 – 0.79)	72 %	100 %	9/50	5/50	5/50	9/50
Synovial hypertrophy	0.65 (0.49 – 0.82)	74 %	100 %	2/50	11/50	11/50	2/50

## Discussion

This study compared the agreement of weight-bearing CBCTA to conventional 3T MRI in evaluating structural imaging pathologies of the knee using the MOAKS system. Cartilage lesions, osteophytes, meniscal pathology, cruciate ligaments, synovial hypertrophy and Baker cysts were evaluated. Whereas the utility of CT arthrogram of the knee has been studied before [[19], [20],^33^,^34^], feasibility studies on CBCTA in evaluating structural knee findings as well as direct comparison studies with MRI using the MOAKS scoring instrument have been scarce [[23], [24], [25]]. We have found both MRI and CBCTA to be very good at depicting intra-articular pathology, although most of our imaging is now done with supine 3T MRI. High-resolution isotropic MRI sequences allow multiplanar reconstruction and better evaluation of previously ambiguous findings, improving the evaluation of small osteophytes, cartilage lesions and especially local hyperintense cartilage signal (MOAKS grade 0), an area where CT has excelled compared to 1.5T MRI [19,23]. MRI is susceptible to metal-induced artifacts, especially with higher field strengths, which is less of an issue with CT. Beam hardening artifacts can induce difficulties in image interpretation in CBCT, and in our experience can sometimes affect the evaluation of retropatellar cartilage surfaces. Using CBCTA as a reference standard, 1.5T MRI was recently reported to have lower diagnostic performance in cartilage lesions in inter-modality comparison [23]. As 3T MRI has been previously shown to perform comparably or better than 1.5T MRI with an arthroscopic reference standard [26], in our current study we evaluated the agreement between weight-bearing CBCTA and supine 3T MRI.

In our results, CBCTA and MRI identified knee joint pathology with moderate to almost perfect agreement. The results were similar to those of a previous investigation comparing spiral CT arthrography, MRI and cadaver histopathology, which reported high sensitivity and specificity for cartilage lesions [19]. In our results, all cartilage lesions demonstrated strong agreement at least between CT and MRI.. The PEAs for full-thickness cartilage lesions were also higher than PEAs in cartilage lesions of all severities in all MOAKS-defined subregions. Whereas small partial cartilage lesions were prevalent in a relatively young and mostly asymptomatic adult population [35], full-thickness cartilage lesions are clearly associated with knee pain [36], and they are central in the Delphi panel definition of MRI-defined knee OA in tibiofemoral as well as patellofemoral joints [31,37]. Although some smaller cartilage lesions can progress over time [36], the identification of full-thickness cartilage lesions in imaging is arguably more important. Where MOAKS grades were discordant, both MRI and CBCTA reported higher cartilage lesion grades than the other in various subregions. Neither modality graded cartilage lesions systematically higher nor produced significantly more false negative findings when the other was considered the reference standard. These results suggest that CBCTA and 3T MRI show very similar variability in their estimation of cartilage lesions.

CBCTA and MRI showed almost perfect agreement in depicting osteophytes. In our study, a high-resolution PD-weighted SPACE sequence was used for MRI, which enables good visualization of even small or equivocal osteophytes. For discordant MOAKS grades, there was an observable tendency for CBCTA to grade osteophytes higher than MRI, although both modalities produced a similar proportion of positive findings where the other modality reported a negative finding. Accordingly, our results suggest that modern isotropic non-fat-saturated MRI sequences offer exceptional identification of osteophytes, comparable to CT [38].

Most meniscal pathologies in the study population were either complex tears or partial macerations. In these meniscal lesions, CBCTA also showed strong agreement at least with MRI. CBCTA performed worst in the medial meniscus body and posterior thirds, where MRI graded fewer meniscal thirds as normal and more as complex tears and macerations. In this regard our results are not as impressive as those previously reported with conventional CT arthrography [20,[39], [40]]; however, we did not pool or categorize imaging findings based on, for example, clinical relevance but focused instead on the comparative agreement with MRI for MOAKS evaluation.

Possibly relating to the high incidence of OA or the high mean age of the study population [41], synovial hypertrophy was seen in 32 % of MRIs and 14 % of CBCTAs with 74 % inter-modality agreement. To our knowledge this is the first study to show that synovial hypertrophy can also be evaluated using CBCTA, as previous publications have focused on articular cartilage lesions, cruciate ligaments and the menisci [[19], [20],^33^,[39], [40]]. Future studies focusing on this “structural” feature are warranted, as for example variability in intra-articular contrast agent concentrations could alter the visibility of amorphous lower HU synovium in CT (Fig. S1). On the other hand, higher concentrations of contrast agent could improve the diagnostics of meniscal lesions.

Certain limitations in our study have to be acknowledged. The sample size of 50 knee joints was relatively low. Images were evaluated by one expert reader, and no intra- or inter-rater data is reported. The reliability of MOAKS has been demonstrated with moderate to high intra- and inter-rater correlation in multiple publications[27,28], but no such data has been published concerning MOAKS evaluation in CBCTA. CT would be expected to provide superior information in features such as subchondral sclerosis and joint space narrowing as well as realistic 3D assessment; however, these are not part of MOAKS and were not evaluated. Eight CBCTAs were technically inadequate and excluded due to absent or very low intra-articular contrast agent concentration preventing diagnostic evaluation; seven were lateral suprapatellar and one a medial tibiofemoral injection. Similar issues have been previously reported [40], and could be mitigated by, for example, postprocedural fluoroscopic evaluation of the knee joint [39] and an optimized CBCTA protocol. Severe patellofemoral or tibiofemoral OA or lack of joint effusion can make injection challenging, which may have affected the choice of injection approach as OA findings in our study population were, as expected [[42], [43], [44]], predominately on the medial side. In some individuals the knee joint space can be exceptionally large, but a standard volume of 10–20 ml was used. The concentration or HU of intra-articular contrast was not standardized or evaluated. CBCTA was not used to evaluate the exact degree of joint effusion as the arthrogram introduced 20 ml of excess fluid into the joint and the volume of joint fluid aspirated before the contrast injection was not recorded. Vertical longitudinal and radial tears were almost completely absent from the study population, and the efficacy of CBCTA in depicting these typically traumatic meniscal lesions could not be evaluated. CT arthrography is reportedly excellent in depicting unstable meniscal tears [39]. An exciting future research prospect would be to repeat the study using supine CBCTA and then to evaluate the added diagnostic value of weight-induced meniscal extrusion in detecting meniscal root tears and other unstable meniscal lesions in posttraumatic patients. This would also enable simultaneous comparison of supine and weight-bearing CBCTA for detecting OA-associated findings. Intra-articular gadolinium in MR arthrography reportedly increases the detection rate of articular cartilage lesions compared to conventional 1.5T MRI [45], but was not evaluated here. Finally, knee arthroscopy as the reference standard would have enabled a simple diagnostic performance evaluation between CBCTA and 3T MRI.

In conclusion, CBCTA is comparable to 3T MRI with moderate to excellent agreement in depicting patellofemoral and tibiofemoral cartilage lesions and osteophytes as well as meniscal lesions, with very good to almost perfect agreement in MOAKS gradings. With optimized protocols, CBCTA offers comparable visualization of OA-associated structural findings with added radioprotective and economic advantages.

**Role of the funding source:** This research received no external funding.

Data available on reasonable request from the authors.

## Declaration of generative AI and AI-assisted technologies in the writing process

Generative AI and AI-assisted technologies were not used in the writing process of this manuscript.

## Declaration of competing interest

The authors declare that they have no known competing financial interests or personal relationships that could have appeared to influence the work reported in this paper.

## Appendix Supplementary materials

Image, application 1

Image, image 2
