# Supplementary material for: Comparative evaluation of weight-bearing cone beam CT arthrography and supine 3T MRI in knee osteoarthritis
Source: Osteoarthr Imaging. 2025 Nov 12;5(4):100382. doi: 10.1016/j.ostima.2025.100382 (PMC13228673; doi:10.1016/j.ostima.2025.100382)
Supplement: Supplementary file 1 [file mmc1.docx]

**Comparative evaluation of weight-bearing cone beam CT arthrography and supine 3T MRI in knee osteoarthritis**

Antti Kemppainen, MD, PhD^a,b*^, Vilja Kotkaranta, MD^a^, Olli Nykänen, PhD^a,c^, Mika T. Nevalainen, MD, PhD^a,b,d^

^a^Research Unit of Health Sciences and Technology

Faculty of Medicine, University of Oulu

P.O. Box 5000, FI-90014 Oulu, Finland

^b^Department of Diagnostic Radiology

Oulu University Hospital

P.O. Box 50, FI-90029 Oulu, Finland

^c^Department of Applied Physics, Faculty of Science and Forestry,

University of Eastern Finland, Kuopio, Finland

^d^Medical Research Center Oulu

University of Oulu and Oulu University Hospital

Oulu, Finland

Declarations of interest: none

Data available on reasonable request from the authors.

ORCID iD:

Antti Kemppainen: 0009-0008-8501-8731

Olli Nykänen: 0000-0001-7329-3463

Mika T. Nevalainen: 0000-0002-9483-7690

*Corresponding author

Antti Kemppainen

Department of Diagnostic Radiology

Oulu University Hospital

P.O. Box 50, FI-90029 Oulu, Finland

E-mail address: antti.kemppainen@pohde.fi

Running headline: Comparison of knee joint imaging findings between cone beam CT arthrography and 3T MRI

**Supplementary data**

**Table S1. Counts and percentages of the CBCTA- and MRI-detected patellofemoral and tibiofemoral cartilage lesions in MOAKS-defined subregions.** Data is presented in an ascending grade severity order (none / <10 % of the cartilage surface area affected / 10-75 % / >75 %). Ant.; Anterior, CBCTA, Cone-beam CT arthrogram, Cent.; Central, Lat.; Lateral, Med.; Medial, MRI; Magnetic resonance imaging, PEA; Percentage of exact agreement, Post.; Posterior.

|  | **CBCTA** |  | **MRI** |  |
| --- | --- | --- | --- | --- |
|  | Cartilage lesions | Full-thickness cartilage lesions | Cartilage lesions | Full-thickness cartilage lesions |
| **Patellofemoral joint** |  |  |  |  |
| Med. patella | 4 / 13 / 16 / 17 | 32 / 11 / 4 / 3 | 5 / 15 / 6 / 24 | 41 / 5 / 0 / 4 |
|  | 8% / 26% / 32% / 34% | 64% / 22% / 8% / 6% | 10% / 30% / 12% / 48% | 82% / 10% / 0% / 8% |
| Lat. patella | 13 / 12 / 13 / 12 | 39 / 6 / 1 / 4 | 12 / 15 / 6 / 17 | 41 / 2 / 4 / 3 |
|  | 26% / 24% / 26% / 24% | 78% / 12% / 2% / 8% | 24% / 30% / 12% / 34% | 82% / 4% / 8% / 6% |
| Ant. med. femur | 7 / 9 / 17 / 17 | 36 / 7 / 5 / 2 | 8 / 8 / 10 / 24 | 34 / 14 / 1 / 1 |
|  | 14% / 18% / 34% / 34% | 72% / 14% / 10% / 4% | 16% / 16% / 20% / 48% | 68% / 28% / 2% / 2% |
| Ant. lat. femur | 11 / 13 / 14 / 12 | 39 / 1 / 4 / 6 | 23 / 6 / 9 / 12 | 40 / 5 / 3 / 2 |
|  | 22% / 26% / 28% / 24% | 78% / 2% / 8% / 12% | 46% / 12% / 18% / 24% | 80% / 10% / 6% / 4% |
| **Tibiofemoral joint** |  |  |  |  |
| Ant. med. tibia | 23 / 2 / 18 / 7 | 39 / 2 / 6 / 3 | 21 / 3 / 12 / 14 | 35 / 1 / 7 / 7 |
|  | 46% / 4% / 36% / 14% | 78% / 4% / 12% / 6% | 42% / 6% / 24% / 28% | 70% / 2% / 14% / 14% |
| Ant. lat. tibia | 42 / 0 / 5 / 3 | 49 / 0 / 1 / 0 | 42 / 0 / 5 / 3 | 48 / 1 / 1 / 0 |
|  | 84% / 0% / 10% / 6% | 98% / 0% / 2% / 0% | 84% / 0% / 10% / 6% | 96% / 2% / 2% / 0% |
| Cent. med. femur | 6 / 7 / 11 / 26 | 23 / 7 / 13 / 7 | 7 / 3 / 4 / 36 | 22 / 5 / 8 / 15 |
|  | 12% / 14% / 22% / 52% | 46% / 14% / 26% / 14% | 14% / 6% / 8% / 72% | 44% / 10% / 16% / 30% |
| Cent. lat. femur | 18 / 5 / 18 / 9 | 34 / 5 / 11 / 0 | 24 / 9 / 4 / 13 | 40 / 0 / 8 / 2 |
|  | 36% / 10% / 36% / 18% | 68% / 10% / 22% / 0% | 48% / 18% / 8% / 26% | 80% / 0% / 16% / 4% |
| Cent. med. tibia | 17 / 4 / 18 / 11 | 35 / 5 / 4 / 6 | 12 / 7 / 8 / 23 | 33 / 2 / 2 / 13 |
|  | 34% / 8% / 36% / 22% | 70% / 10% / 8% / 12% | 24% / 14% / 16% / 46% | 66% / 4% / 4% / 26% |
| Cent. lat. tibia | 31 / 5 / 7 / 7 | 41 / 2 / 6 / 1 | 23 / 11 / 7 / 9 | 40 / 0 / 5 / 5 |
|  | 62% / 10% / 14% / 14% | 82% / 4% / 12% / 2% | 46% / 22% / 14% / 18% | 80% / 0% / 10% / 10% |
| Post. med. femur | 13 / 5 / 20 / 12  26% / 10% / 40% / 24% | 33 / 5 / 10 / 2  66% / 10% / 20% / 4% | 17 / 4 / 15 / 14  34% / 8% / 30% / 28% | 37 / 10 / 3 / 0  74% / 20% / 6% / 0% |
| Post. lat. femur | 21 / 9 / 11 / 9 | 38 / 3 / 8 / 1 | 28 / 4 / 9 / 9 | 42 / 3 / 4 / 1 |
|  | 42% / 18% / 22% / 18% | 76% / 6% / 16% / 2% | 56% / 8% / 18% / 18% | 84% / 6% / 8% / 2% |
| Post. med. tibia | 19 / 7 / 21 / 3 | 41 / 2 / 6 / 1 | 22 / 8 / 16 / 4 | 40 / 2 / 7 / 1 |
|  | 38% / 14% / 42% / 6% | 82% / 4% / 12% / 2% | 44% / 16% / 32% / 8% | 80% / 4% / 14% / 2% |
| Post. lat. tibia | 24 / 9 / 10 / 7 | 39 / 3 / 6 / 2 | 23 / 12 / 7 / 8 | 40 / 0 / 3 / 7 |
|  | 48% / 18% / 20% / 14% | 78% / 6% / 12% / 4% | 46% / 24% / 14% / 16% | 80% / 0% / 6% / 14% |

**Table S2. Counts and percentages of the CBCTA- and MRI-detected patellofemoral and tibiofemoral osteophytes in MOAKS-defined subregions.** Data is presented in an ascending grade severity order (none / small / moderate / large osteophyte). CBCTA, Cone-beam CT arthrogram, MRI; Magnetic resonance imaging.

|  | **CBCTA** | **MRI** |
| --- | --- | --- |
|  |  |  |
| **Patellofemoral joint** |  |  |
| Superior patella | 5 / 19 / 20 / 6 | 9 / 24 / 11 / 6 |
|  | 10% / 38% / 40% / 12% | 18% / 48% / 22% / 12% |
| Inferior patella | 8 / 14 / 21 / 7 | 14 / 12 / 18 / 6 |
|  | 16% / 28% / 42% / 14% | 28% / 24% / 36% / 12% |
| Medial patella | 11 / 14 / 19 / 6 | 8 / 16 / 25 / 1 |
|  | 22% / 28% / 38% / 12% | 16% / 32% / 50% / 2% |
| Lateral patella | 4 / 16 / 22 / 8 | 4 / 19 / 25 / 2 |
|  | 8% / 32% / 44% / 16% | 8% / 38% / 50% / 4% |
| Medial trochlea | 11 / 5 / 23 / 11 | 10 / 10 / 17 / 13 |
|  | 22% / 10% / 46% / 22% | 20% / 20% / 34% / 26% |
| Lateral trochlea | 14 / 14 / 14 / 8 | 17 / 9 / 17 / 7 |
|  | 28% / 28% / 28% / 16% | 34% / 18% / 34% / 14% |
| **Tibiofemoral joint** |  |  |
| Posterior medial femur | 2 / 8 / 24 / 16 | 2 / 11 / 21 / 16 |
|  | 4% / 16% / 48% / 32% | 4% / 22% / 42% / 32% |
| Posterior lateral femur | 10 / 14 / 16 / 10 | 11 / 14 / 19 / 6 |
|  | 20% / 28% / 32% / 20% | 22% / 28% / 38% / 12% |
| Central medial femur | 7 / 4 / 15 / 24 | 6 / 8 / 18 / 18 |
|  | 14% / 8% / 30% / 48% | 12% / 16% / 36% / 36% |
| Central lateral femur | 10 / 7 / 10 / 23 | 9 / 13 / 14 / 14 |
|  | 20% / 14% / 20% / 46% | 18% / 26% / 28% / 28% |
| Central medial tibia | 5 / 7 / 20 / 18 | 7 / 8 / 22 / 13 |
|  | 10% / 14% / 40% / 36% | 14% / 16% / 44% / 26% |
| Central lateral tibia | 13 / 10 / 14 / 13 | 15 / 13 / 16 / 6 |
|  | 26% / 20% / 28% / 26% | 30% / 26% / 32% / 12% |

**Table S3. Counts and percentages of the CBCTA- and MRI-detected meniscus morphology, cruciate ligament, Baker’s cyst and synovial hypertrophy findings.** Grades are presented in an ascending order (for the menisci: normal / intrameniscal signal / vertical tear / horizontal tear / complex tear / partial maceration / total maceration; for ACL and PCL: intact / torn; for Baker’s cyst and synovial hypertrophy: absent / present). ACL; Anterior cruciate ligament, CBCTA; Cone-beam CT arthrography, MRI; Magnetic resonance imaging, PCL; Posterior cruciate ligament.

|  | CBCTA | MRI |
| --- | --- | --- |
| Anterior medial meniscus | 41 / 0 / 0 / 0 / 2 / 7 / 0  82% / 0% / 0% / 0% / 4% / 14% / 0% | 47 / 0 / 0 / 0 / 0 / 3 / 0  94% / 0% / 0% / 0% / 0% / 6% / 0% |
| Medial meniscus body | 30 / 0 / 0 / 1 / 8 / 11 / 0  60% / 0% / 0% / 2% / 16% / 22% / 0% | 23 / 1 / 0 / 2 / 11 / 12 / 1  46% / 2% / 0% / 4% / 22% / 24% / 2% |
| Posterior medial meniscus | 28 / 0 / 0 / 4 / 10 / 8 / 0  56% / 0% / 0% / 8% / 20% / 16% / 0% | 19 / 1 / 1 / 4 / 7 / 17 / 1  38% / 2% / 2% / 8% / 14% / 34% / 2% |
| Anterior lateral meniscus | 43 / 0 / 1 / 0 / 0 / 6 / 0  86% / 0% / 2% / 0% / 0% / 12% / 0% | 42 / 0 / 0 / 1 / 1 / 5 / 1  84% / 0% / 0% / 2% / 2% / 10% / 2% |
| Lateral meniscus body | 43 / 0 / 0 / 1 / 1 / 5 / 0  86% / 0% / 0% / 2% / 2% / 10% / 0% | 38 / 0 / 0 / 2 / 4 / 2 / 4  76% / 0% / 0% / 4% / 8% / 4% / 8% |
| Posterior lateral meniscus | 40 / 0 / 0 / 1 / 5 / 2 / 2  80% / 0% / 0% / 2% / 10% / 4% / 4% | 43 / 0 / 0 / 0 / 1 / 4 / 2  86% / 0% / 0% / 0% / 2% / 8% / 4% |
| ACL tear | 47 / 3 | 48 / 2 |
|  | 94% / 6% | 96% / 4% |
| PCL tear | 50 / 0 | 50 / 0 |
|  | 100% / 0% | 100% / 0% |
| Baker’s cyst | 26 / 24 | 22 / 28 |
|  | 52% / 48% | 44% / 56% |
| Synovial hypertrophy | 7 / 43  14% / 86% | 16 / 34  32% / 68% |


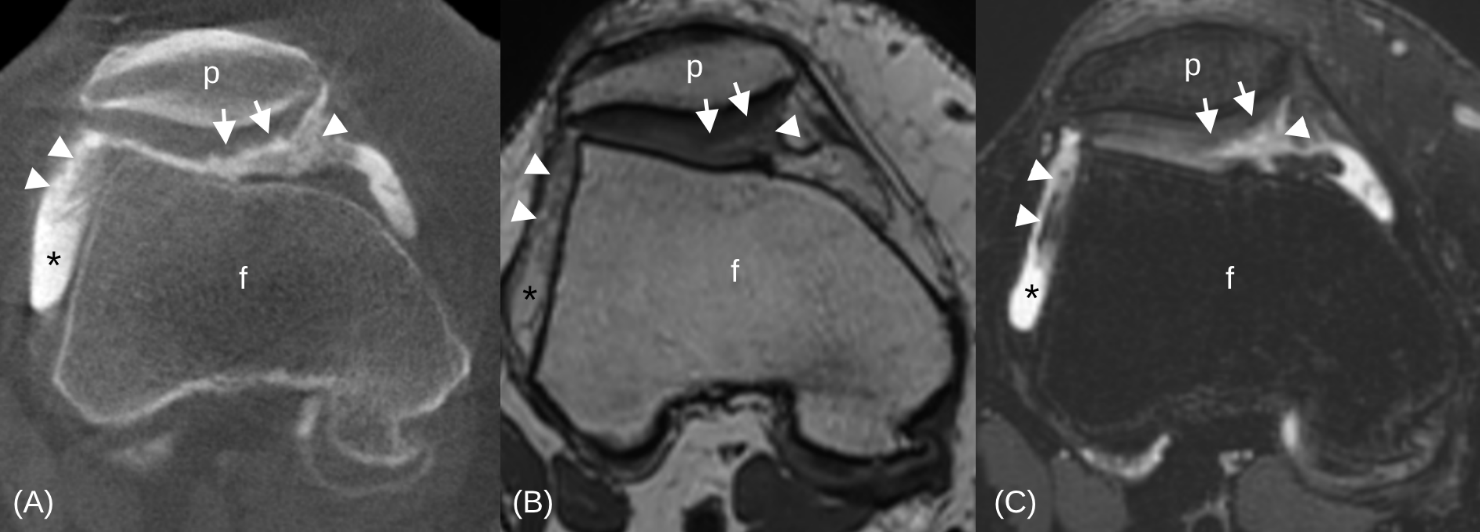

Figure S1. A patellofemoral cartilage lesion and synovial hypertrophy in a 57-year-old woman in axial orientation. The cartilage lesion of the lateral patellar facet (arrows) is outlined in CBCTA (A) similarly to MRI (B and C). In addition, synovial hypertrophy is seen as amorphous areas of lower Hounsfield unit value material (arrowheads) in the joint effusion (asterisks) similarly to PD- (B) and T2-weighted fat saturated (C) MRI. The patient also had severe medial tibiofemoral osteoarthritis that was thought to be the cause of the synovial hypertrophy and effusion shown here. f; femur, p; patella.
