# Supplementary figures and images for: Comparative evaluation of weight-bearing cone beam CT arthrography and supine 3T MRI in knee osteoarthritis
Source: Osteoarthr Imaging. 2025 Nov 12;5(4):100382. doi: 10.1016/j.ostima.2025.100382 (PMC13228673; doi:10.1016/j.ostima.2025.100382)

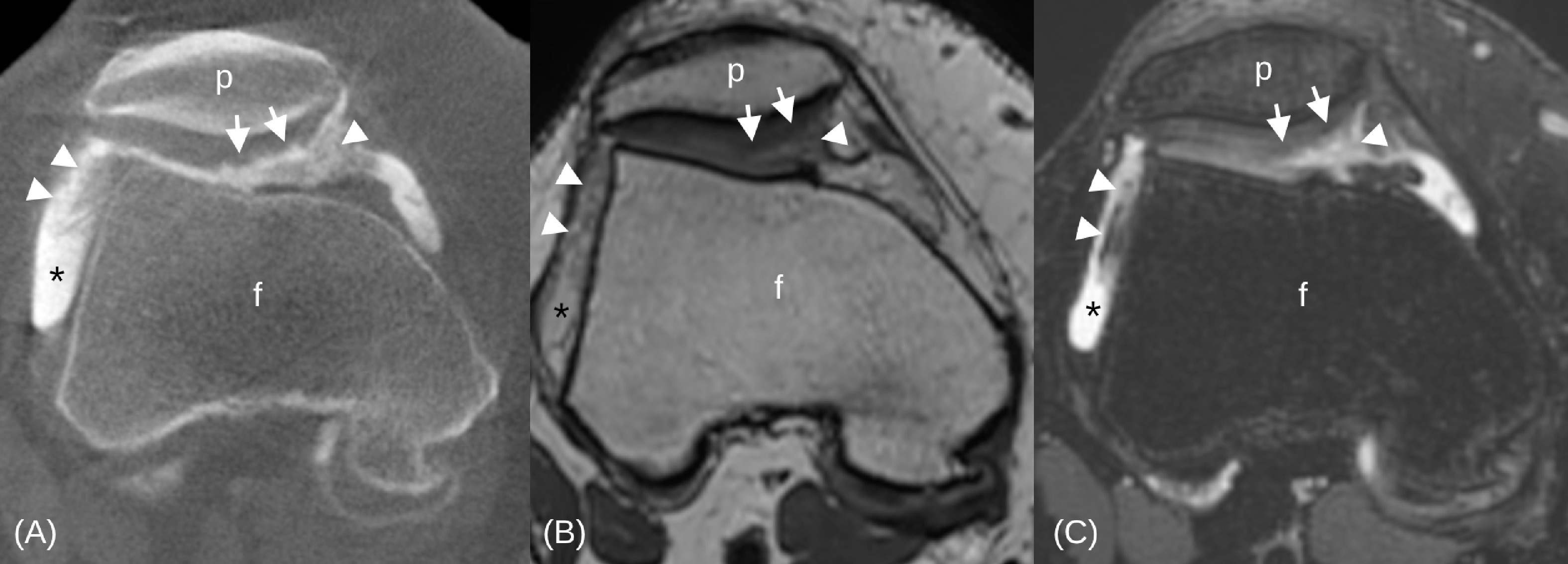

Supplement: Supplementary file 2 [file mmc2.jpg]
